# A Rare Case of Pseudo-Meigs' Syndrome With Ovarian Metastasis Presenting as Meigs' Syndrome

**DOI:** 10.7759/cureus.11022

**Published:** 2020-10-18

**Authors:** Nimit Dalal, Pal Satyajit Singh Athwal, Biswaraj Tharu, Lakshmi Saravanan, Hassan Mansour

**Affiliations:** 1 Internal Medicine, Trumbull Regional Medical Center, Warren, USA; 2 Internal Medicine, Saraswathi Institute of Medical Sciences, Hapur, IND; 3 Internal Medicine, American University of Antigua School of Medicine, New York, USA

**Keywords:** meigs´ syndrome, pseudo-meigs’, ovarian cancer

## Abstract

Pseudo-Meigs' syndrome is defined as malignant ovarian tumor leading to ascites or/and pleural effusion, whereas Meigs' syndrome is a triad of ascites, pleural effusion, and benign ovarian tumor. The removal of an underlying tumor leads to rapid improvement in patient symptoms in both conditions. It is a rare phenomenon, and only 1% of ovarian tumors account for Meigs' syndrome. We report a case of a 70-year-old female presented with complaints of shortness of breath, vaginal bleeding, bloating, and increased abdominal girth. X-ray and lab workup revealed pleural effusion and raised CA 125 (cancer antigen 125), which along with clinical presentation raised suspicion for Meigs' syndrome, but on exploratory laparotomy ovarian serous carcinoma was diagnosed. Diagnosis of pseudo-Meigs' syndrome was established instead of Meigs' syndrome, which was initially suspected. Pseudo-Meigs' syndrome can mimic many other pathologies, which makes it a diagnostic challenge.

## Introduction

In 1937, Meigs and Cass first reported case series of patients with ascites, pleural effusion, and ovarian fibroma [1]. There has been much discrepancy about the exact definition of Meigs' syndrome. Based on case reports, review articles, and various studies available, the following four criteria should be met to classify a case as classic Meigs' syndrome:

1) Benign ovarian fibroma

2) Ascites

3) Pleural effusion

4) Resolution of ascites and pleural effusion after tumor removal [2]

However, some authors like to describe any case of low-grade ovarian malignant tumor as associated with ascites and pleural effusion that disappear after removal of the tumor as Meigs' syndrome. On the other hand, pseudo-Meigs' syndrome is a rare neoplastic disease characterized by the presence of a benign or malignant pelvic or abdominal tumor (other than ovarian fibroma or fibroma-like tumor) associated with hydrothorax and ascites that resolve after tumor resection [3]. Here we present a unique case of pseudo-Meigs' syndrome mimicking Meigs' syndrome on initial presentation, but on further investigation it turned out to be a high-grade ovarian serous carcinoma with metastasis to lymph nodes and uterine myometrium.

## Case presentation

**Figure 1:**
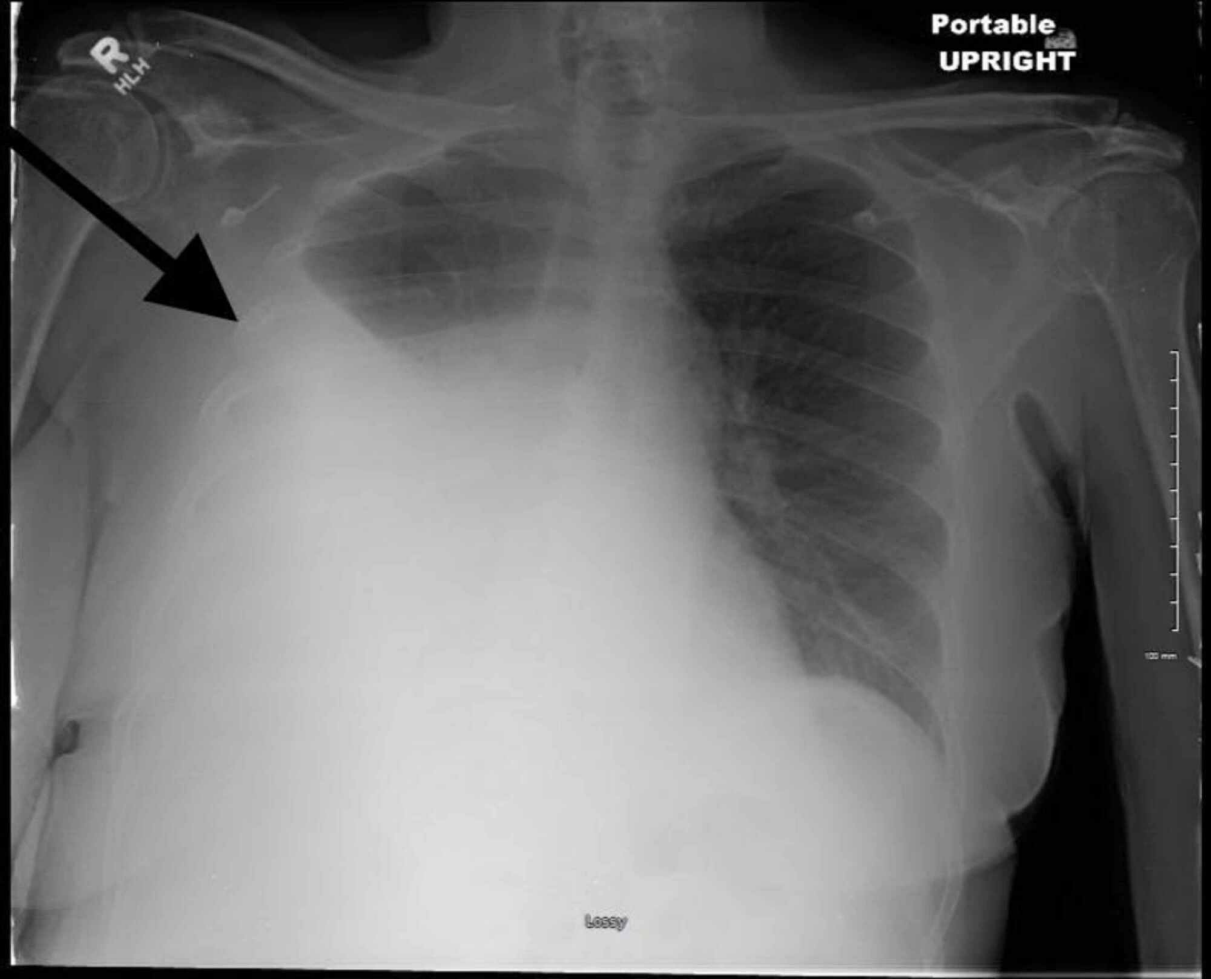
Chest X-ray showing large right-sided pleural effusion.

A 72-year-old female with a past medical history of hypertension, hyperlipidemia, and type 2 diabetes was admitted to our department with the chief complaint of shortness of breath since a week. Symptoms were present at rest as well as with exertion. The patient also complained of increase in abdominal girth and bloating over the last two months. One month prior to presentation, she was seen by a gynecologist for vaginal bleeding. Ultrasonography of the abdomen and pelvis was performed, which showed a pelvic mass. The patient missed the follow-up appointment with the gynecologist. The patient denied any joint pain, peripheral edema, or history of liver or cardiovascular disease. On respiratory examination, there was right-sided dullness with absent breath sounds, and abdominal examination revealed shifting dullness on percussion. Chest X-ray (Figure 1) on admission showed a large right-sided pleural effusion.

Pulmonology service was consulted, and thoracentesis was performed, during which 1,500 cc of amber color fluid was obtained. Pleural fluid analysis was performed, as shown in Table 1.

**Table 1:** Results of pleural fluid analysis WBC, white blood cells; RBC, red blood cell; LDH, lactate dehydrogenase

Pleural Fluid Analysis	
Color	Yellow
Appearance	Cloudy
WBC	1,943
RBC	8,000
Polymorphonuclear leukocytes %	28
Lymphocytes %	14
Mesothelial %	19
Total protein	5.2
LDH	340

**Figure 2:**
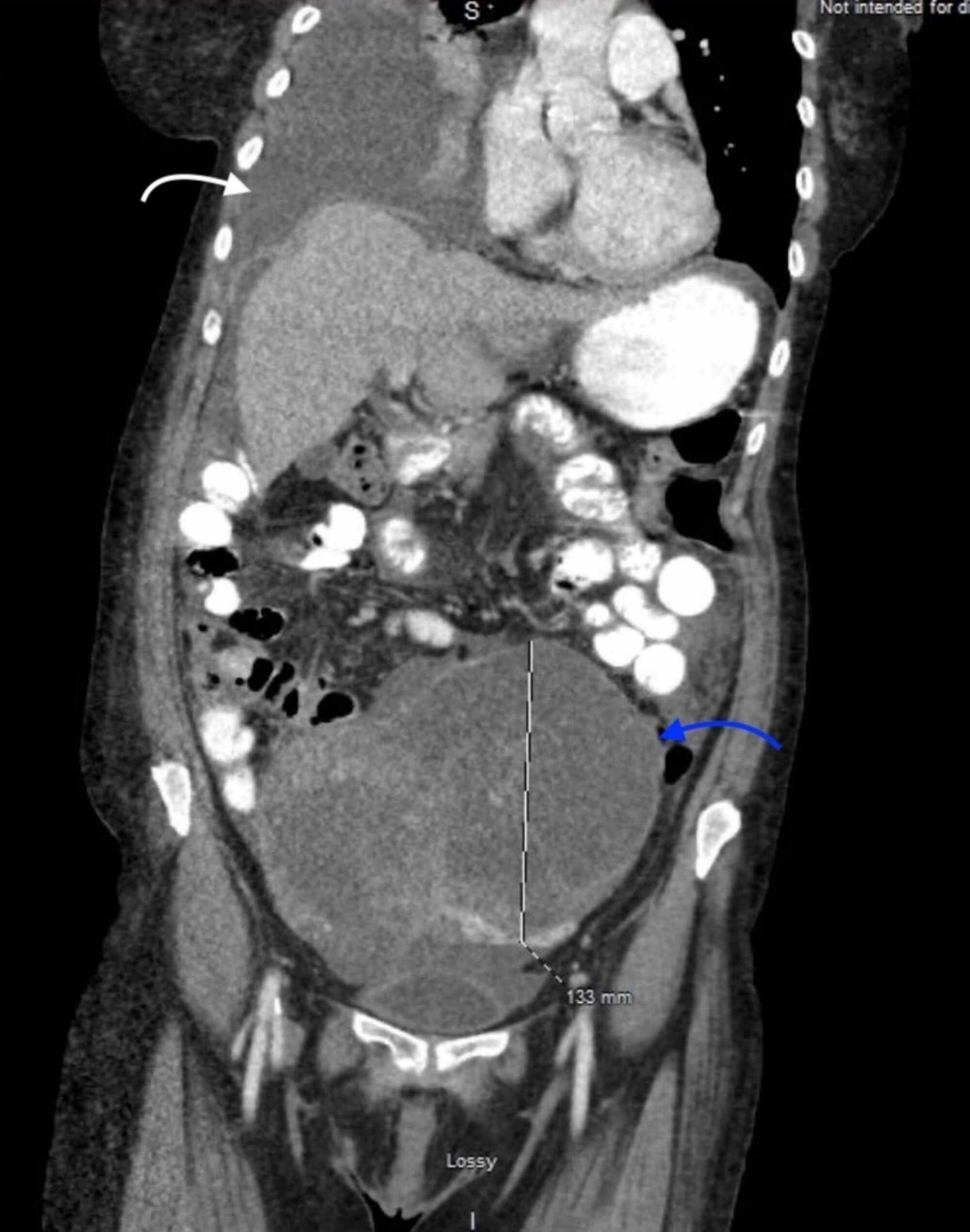
CT scan showing pleural effusion (white arrow) and a large pelvic mass (blue arrow).

Meigs' syndrome was suspected because of presentation as well as lab workup. A right PleurX® catheter (Becton, Dickinson and Company, Franklin Lakes, NJ, USA) was placed on pulmonology recommendations. However, on day 3 of admission, the patient’s cancer antigen 125 (CA 125) level was 3,653. A CT scan demonstrated pelvic mass along with pleural effusion and ascites (Figure 2).

Ovarian malignancy was suspected, and the patient was transferred to a tertiary care hospital for further workup. An exploratory laparotomy was performed, which showed high-grade ovarian serous carcinoma, with the greatest dimension being 17.5 cm with architectural features suggestive of solid endometrial-like transitional variant after total hysterectomy and bilateral salpingo-oophorectomy. The postoperative course was uneventful, and the PleurX® catheter was removed on the third postoperative day. Postoperative diagnoses were ovarian malignancy, secondary malignancy of the omentum, and pelvic peritoneum and pelvic lymph nodes.

After surgery, the pleural effusion and ascites resolved. The patient is scheduled to receive six courses of carboplatin and taxols.

## Discussion

The combination of ascites, pleural effusion, and ovarian mass in an elderly lady with elevated CA 125 raises the suspicion of potential ovarian malignancy. While there is a correlation between CA 125 and ovarian malignancy, an elevated CA 125 has been often noted on some benign cases of Meigs' syndrome. CA 125 is a non-specific tumor marker and should not be used in making a diagnosis of ovarian malignancy. Many other conditions also cause elevated CA 125 level, including endometriosis, liver cirrhosis, pelvic inflammatory disease, and uterine fibroids.

Several theories have been suggested to explain the origin of ascites and pleural effusion in Meigs' syndrome and pseudo-Meigs' syndrome; however, the exact cause still remains unclear, although it appears to be related to lymphatic obstructions. Pressure on the lymphatics from the tumor may result in escape of fluid through surface lymphatics [4,5].

A combination of leakage of intratumoral fluid, mechanical irritation from the tumor, and peritoneal inflammation may result in the production of ascites. In regards to pleural fluid, mechanical transfer of ascitic fluid through the diaphragmatic apertures or through lymphatics has been suggested [5-8].

It has been suggested that because transdiaphragmatic lymphatic channels are larger in diameter on the right, the pleural effusion in Meigs' syndrome `and pseudo-Meigs' syndrome is on the right side too. However, left-sided pleural effusions and bilateral pleural effusions have been reported [3]. It can presents as right-sided pleural effusion along with ascites, as seen in our patient, though rarely pericardial effusion has been reported in the past and termed Meigs'-like syndrome [9]. Diagnosis involves ruling out other condition based on pleural/ascitic fluid analysis and imaging. Pseudo-pseudo-Meigs' syndrome has been reported in patients with systemic lupus erythematosus, which should be ruled out with appropriate serology testing [10].

Prognosis of this condition is very favorable, and removal of underlying tumor leads to resolution of both ascites and pleural effusion within a week. In our patient, symptoms resolved following the surgery.

## Conclusions

In conclusion, Meigs' syndrome and pseudo-Meigs' syndrome cannot be differentiated from a clinical perspective and require further pathological studies. While used to monitor therapeutic progress, CA125 serum levels are a poor biochemical marker used in the diagnosis and differentiation of malignant versus benign ovarian mass. This is a unique case of pseudo-Meigs' syndrome mimicking Meigs' syndrome on presentation, but on further investigation it turned out be an ovarian adenocarcinoma. The patient underwent total hysterectomy with bilateral salpingo-oophorectomy, and the ascites and pleural effusion resolved. The list of differentials in such cases is never-ending, and underlying tumor that might be responsible for such presentation should always be kept in mind.
